# Antigenic Evolution on a Global Scale Reveals the Potential Natural Selection of Severe Acute Respiratory Syndrome-Coronavirus 2 by Pre-existing Cross-Reactive T-Cell Immunity

**DOI:** 10.3389/fmicb.2021.599562

**Published:** 2021-05-18

**Authors:** Chengdong Zhang, Xuanxuan Jin, Xianyang Chen, Li Qiu, Qibin Leng, Tianyi Qiu

**Affiliations:** ^1^Shanghai Public Health Clinical Center, Fudan University, Shanghai, China; ^2^State Key Laboratory of Respiratory Diseases, Affiliated Cancer Hospital & Institute of Guangzhou Medical University, Guangzhou, China; ^3^Department of Clinical Oncology, Taihe Hospital, Hubei University of Medicine, Shiyan, China

**Keywords:** severe acute respiratory syndrome-coronavirus 2, pre-existing cross-reactive T-cell immunity, vaccine design, antigenic evolution, seasonal human coronavirus

## Abstract

The mutation pattern of severe acute respiratory syndrome-coronavirus 2 (SARS-CoV-2) has changed constantly during worldwide community transmission of this virus. However, the reasons for the changes in mutation patterns are still unclear. Accordingly, in this study, we present a comprehensive analysis of over 300 million peptides derived from 13,432 SARS-CoV-2 strains harboring 4,420 amino acid mutations to analyze the potential selective pressure of the host immune system and reveal the driver of mutations in circulating SARS-CoV-2 isolates. The results showed that the nonstructural protein ORF1ab and the structural protein Spike were most susceptible to mutations. Furthermore, mutations in cross-reactive T-cell epitopes between SARS-CoV-2 and seasonal human coronavirus may help SARS-CoV-2 to escape cellular immunity under long-term and large-scale community transmission. Additionally, through homology modeling and protein docking, mutations in Spike protein may enhance the ability of SARS-CoV-2 to invade host cells and escape antibody-mediated B-cell immunity. Our research provided insights into the potential mutation patterns of SARS-CoV-2 under natural selection, improved our understanding of the evolution of the virus, and established important guidance for potential vaccine design.

## Introduction

The coronavirus disease 2019 (COVID-19) epidemic caused by severe acute respiratory syndrome-coronavirus 2 (SARS-CoV-2) is now a worldwide pandemic (Lu et al., 2020; Tang et al., 2020). The whole-genome sequence of SARS-CoV-2 was first released in January 2020 (Wu et al., 2020; Zhou et al., 2020), followed by detection of massive strain isolates from human patients (Shu and McCauley, 2017). Analysis of whole-genome sequences (Shu and McCauley, 2017; Lu et al., 2020) has shown that mutations were already present in both structural and nonstructural proteins of SARS-CoV-2, leading to the emergence of different subtypes (Tang et al., 2020) and affecting the pathogenicity of SARS-CoV-2 (Yao et al., 2020). However, the potential drivers of these mutations have not been identified, and comprehensive investigations are needed to analyze the evolutionary pressure and virulence of the circulating SARS-CoV-2 isolates.

The evolution of viruses is mainly affected by large-scale transmission among the host population, which could provide opportunities for mutant strains to selectively thrive under evolutionary pressure by the host immune system, including both humoral immunity and cellular immunity (Petrova and Russell, 2018). Unlike humoral immunity, which primarily targets the main antigen protein of the virus, T-cell-mediated cellular immunity can respond to viral infection by recognition of human leukocyte antigen (HLA) restriction, in which the linear epitope from the whole protein can be presented by HLA (Kindt et al., 2007). HLA alleles vary according to geography and ethnicity in populations around the world. Therefore, because of HLA allelic diversity and gene polymorphisms, which are frequently associated with susceptibility to viral infections (Setiawan et al., 2015; Karimzadeh et al., 2019), T-cells may respond differently to the same antigen. HLA diversity at population-level may also have driven the rapid evolution of SARS-CoV-2 during its global spread in the past 6 months based on variations in HLA-restricted T-cell immunity.

Including SARS-CoV-2, which is a newly emerging pathogen, seven human coronaviruses (HCoVs) have also been reported. Among these viruses, HCoV-229E, HCoV-HKU1, HCoV-NL63, and HCoV-OC43 are seasonal HCoVs that have been circulating in the community for a long time and only cause slight respiratory symptoms (Gaunt et al., 2010). Moreover, multiple studies have suggested the existence of cross-reactive T-cell recognition between circulating seasonal HCoVs and SARS-CoV-2 (Bert et al., 2020; Grifoni et al., 2020; Kissler et al., 2020). By accessing genome sequence data from the worldwide spread of SARS-CoV-2, it may be possible to identify the mechanisms of selective pressure mediated by the immune system and the potential evolutionary direction of SARS-CoV-2.

**FIGURE 1:**
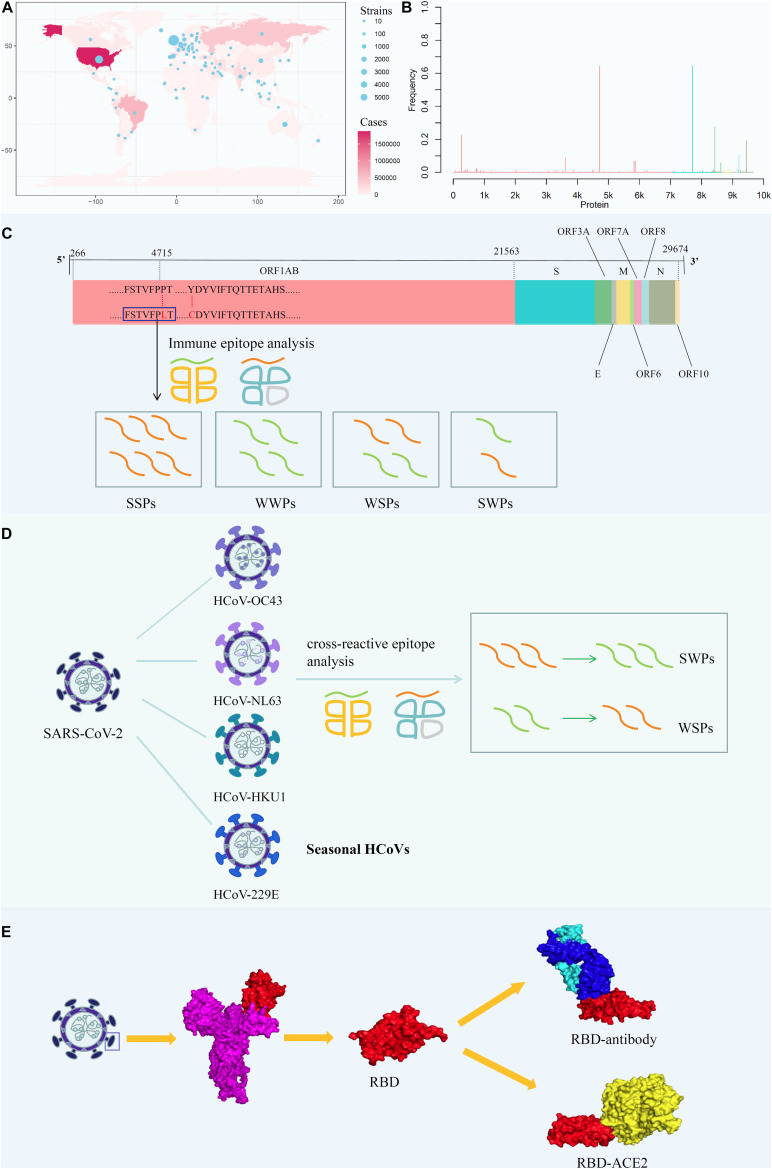
Analysis the mutation pattern of the SARS-CoV-2 whole genome. **(A)** Collecting mutation strains of SARS-CoV-2 in worldwide scale. **(B)** Mapping the mutations on the whole genome sequence of SARS-CoV-2. **(C)** Deriving potential T-cell epitopes involving mutations in different structures of the whole genome sequence. According to the binding affinity predicted by IEDB standalone tools, the peptides were divided into strong binding peptide and weak binding peptide. If the mutation shifted the binding affinity of the peptides from strong to weak, it will be counted as SWPs, similarly, WSPs represents weak to strong peptides. If the binding affinity remains the same, it will be marked as SSPs and WWPs. **(D)** Revealing the selective pressure of cross-reactive epitopes between seasonal HCoVs and SARS-CoV-2 according to the circulating regions of viruses and the local dominant alleles. Here, by use local blast, the peptides with the same sequence between SARS-CoV-2 and other four common HCoVs were derived as cross-reactive epitopes. **(E)** Evaluating the binding affinity of S protein mutants against human ACE2 and binding antibody CR3022. The second subgraph is the crystal structure of SARS-CoV-2 S protein. The third subgraph is the crystal structure of the RBD region. Then, the binding complexes of RBD-ACE2 and RBD-mAb were also demonstrated.

Accordingly, in this study, we conducted a comprehensive analysis, including mapping amino acid mutations on the whole-genome sequence of SARS-CoV-2 and screening all potential T-cell epitopes (PTEs) involving mutation sites (Figures 1A,B); analyzing the immunogenicity of potential peptides based on the circulating regions of viruses worldwide and local dominant alleles (Figure 1C); analyzing the selective pressure of HLA through cross-reactive epitopes (CREs) between seasonal HCoVs and SARS-CoV-2 (Figure 1D); and evaluating the binding affinity of S protein mutants against human angiotensin-converting enzyme 2 (ACE2) and binding antibodies (Figure 1E).

## Frequent Mutations on ORF1ab and Spike (S) Proteins From SARS-CoV-2

**FIGURE 2:**
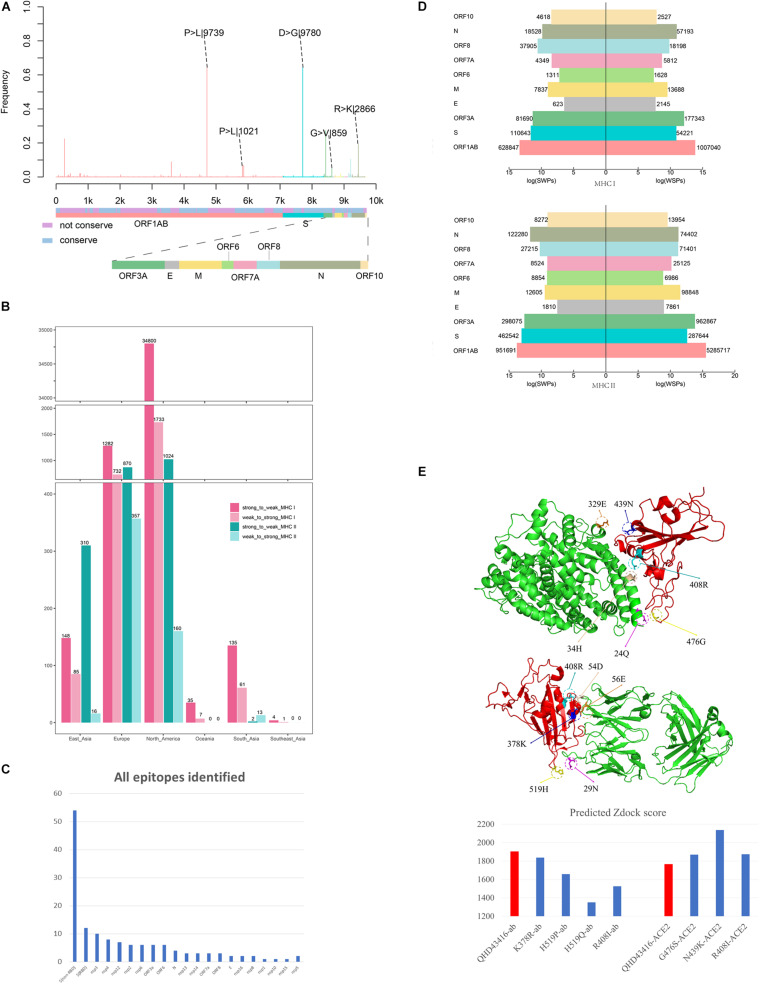
Mutation profile and potential selective pressure analysis of SARS-CoV-2. **(A)** Mutation patterns on the whole genome of SARS-CoV-2. The bar plot shows the frequency of every site in the genome sequence, as different colors representing different structures of the genome sequence. **(B)** Number of SWPs and WSPs on the whole genome of SARS-CoV-2. The bar plot shows number of SWPs and WSPs in different structures of the sequence. SWPs represents strong to weak peptides; WSPs represents weak to strong peptides. **(C)** Number of all epitopes identified in Matenus’s work. The bar plot shows the number of all epitopes identified in Matenus’s work. ORF1ab is cleaved into many nonstructural proteins (NSP1-NSP16). S(RBD) represents receptor-binding domain in spike protein; S(non-RBD) represents the non-RBD portion of spike. N:nucleocapsid protein; E:envelope protein; ORF: open reading frame; **(D)** Number of cross-reactive SWPs and WSPs on ORF1ab. The bar plot shows number of CREs for HLA-I and HLA-II on all 6 continents including North America, Europe, South Asia, East Asia, Southeast Asia, and Oceania. CREs: cross-reactive epitopes. HLA:human leukocyte antigen **(E)** Impact of binding affinity for mutations on the S protein. The plot in left shows the 3-D structure changes in the mutation sites, and the bar plot in the right shows predicted Zdock score of the mutation site between RBD and ACE2.

We observed 4,420 amino acid mutation sites on 10 structural and nonstructural proteins, including ORF1ab, S, ORF3a, envelope (E), membrane (M), ORF6, ORF7a, ORF8, nucleocapsid (N), and ORF10 (Figure 2A). The results showed that ORF1ab, S, ORF3a, M, ORF7a, ORF8, and N contained both conserved and non-conserved regions. Among these proteins, ORF1ab and S proteins showed the highest frequency of mutation sites, with a mutation frequency of greater than 0.6 (Figure 2A). The mutation frequency and counts of each residue are described in Supplementary Table 1. Furthermore, we counted the continent-specific mutation sites and identified the most frequent mutations occurring on S, ORF1ab, N, and ORF3a proteins. Among the top five mutations, D614G on S protein was counted 9,780 times, followed by P4715L on ORF1ab (9,745 times), R203K on N protein (2,866 times), P5828L on ORF1ab (1,021 times), and G251V on ORF3a (859 times). Detailed information for top frequency mutation sites can be found in Supplementary Table 2. Interestingly, the most frequently mutated sites were detected from strains circulating in Europe and Northern America, which have become major epicenters of the epidemic; these findings indicated that the immune pressure could have contributed to virus evolution. For example, the D614G mutation on S protein and the P4715L mutation on ORF1ab protein were counted approximately 3,000 times in the United States of America and the United Kingdom (Supplementary Table 3). Currently, countries or regions such as the United States, and continental Europe are suffering from the epidemic and are among high-incidence areas of SARS-CoV-2. It can be observed that the long-term and large-scale community transmission in these countries makes high mutation frequency in both ORF1ab and S protein on the whole genome of SARS-CoV-2 (Mercatelli and Giorgi, 2020). The frequent mutations observed on S protein and ORF1ab may be related to the selective pressure of humoral immunity and cellular immunity after the long-term, large-scale community transmission of SARS-CoV-2.

Moreover, using the immunogenicity prediction analysis tool through T-cell class I pMHC immunogenicity predictor, the results of epitopes involving mutations in ORF1ab can be found in Supplementary Table 4. Results illustrated that, among 86 9-mer epitopes involving mutations in ORF1ab, 36 epitopes achieved positive scores, which indicates the ability to influence immunogenicity in T-cell immunity. Also, using the predicted epitopes involving mutations in our work, it can be found that peptide LAILTALRLCAYCCN was identified as T-cell epitopes for HLA alleles DRB1^∗^01:01 and DRB1^∗^11:01. This result is consistent in both our *in-silico* research and the most recent experimental research of Matenus J’s work (Mateus et al., 2020), which indicating the predicted T-cell epitopes involving mutations could actually inducing T-cell responses.

## Mutations Shifted the Immunogenicity of T-Cell Peptides

With the worldwide spread of COVID-19, the numbers of infections and affected nations or regions have continued to grow. We performed epitope prediction based on the tools provided by the Immune Epitope Database (IEDB) to select epitopes with 8-mer to 15-mer peptides for each strain; 366,286,292 peptides were obtained, and 4,420 amino acid mutations derived from 13,432 SARS-CoV-2 isolates were observed. For each strain with location information, the HLA alleles with the dominant population coverage (allele frequency ≥ 0.05) were selected to predict the binding affinity of peptides through IEDB stand-alone tools (Moutaftsi et al., 2006; Wang et al., 2008). The reference sequence of SARS-CoV-2 (MN908947.3) was selected for comparison. The results showed that most of the mutations on PTEs did not change the predicted binding affinity (Supplementary Tables 5, 6). For mutations that altered binding affinity, peptides changing from weak binding to strong binding (WSPs) predominated over those changing from strong binding to weak binding (SWPs). As illustrated in Figure 2B, WSPs outnumbered SWPs for both HLA-I and HLA-II PTEs on most of the nonstructural and structural proteins. In contrast, for S protein, SWPs accounted for 2.2 and 1.8 times the number of WSPs for both HLA-I and HLA-II PTEs, respectively.

## HLA-Mediated Immune Pressure Promoted the Evolution of SARS-CoV-2

Infection by seasonal HCoVs is ubiquitous and causes minor symptoms, similar to those of the common cold (Lidwell and Williams, 1961; Tyrrell, 1965; Gaunt et al., 2010). Epidemiological data have suggested that HCoVs can infect adults every 2–3 years (Braun et al., 2020). Continuous transmission of HCoVs in the human population enables maintenance of immune memory and produces selective pressure for CREs between HCoVs and SARS-CoV-2. To explore the roles of memory T-cell immunity to HCoVs on SARS-CoV-2 evolution, we analyzed the CREs between SARS-CoV-2 and HCoVs that may have already circulated throughout the community before the COVID-19 epidemic. A recent study of Matenus J’s work (Mateus et al., 2020) proved that cross-reactive SARS-CoV-2 T-cell epitopes can be detected in unexposed humans, which indicating the variegated T-cell memory to coronaviruses that cause the common cold may underlie at least some of the extensive heterogeneity observed in COVID-19 disease. Here, we mapped the T-cell epitopes in Matenus J’s work in the whole genome to illustrate the distribution of epitopes that involving mutations (Supplementary Table 7 and Figure 2C). It can be found that epitopes are mostly enriched in the S protein of the whole SARS-CoV-2 genome, including both the non-RBD and RBD regions. This is consistent with our results that besides ORF1ab, S protein contains the most T-cell epitopes with mutations (Supplementary Table 1). The presentation of T-cell epitopes significantly associated with the HLA types (Hoof et al., 2009). Thus, before the prediction of binding affinity between peptides and MHC molecules, the HLA alleles were derived from Allele Frequency Net Database (Gonzalez-Galarza et al., 2020) and the major HLA alleles in different regions were screened based on frequency. For each region, the HLA alleles recorded in over 5% of the local populations were derived for further analysis, which can be found in Supplementary Table 8. By matching information from original infectious regions with the dominant HLA-I/HLA-II alleles, 197/358 alleles and 9,327/943 peptides from 13/11 nations in 7/4 continents, respectively, were obtained for further analysis. For each CRE, the binding affinities of HLA-I/HLA-II with the corresponding alleles were predicted by IEBD MHC-I/MHC-II binding prediction tools.

The number of WSPs was 1.6 times that of SWPs on ORF1ab protein for all peptides involving mutations (Supplementary Table 9). However, analysis of the number of CREs revealed the opposite results, in which cross-reactive SWPs were significantly more frequent than cross-reactive WSPs on six continents, i.e., North America, Europe, South Asia, East Asia, Southeast Asia, and Oceania (Figure 2C). For example, the results in North America showed that cross-reactive HLA-I SWPs (34,800) were 20.1 times as frequent as cross-reactive WSPs (1,733). Similar results were also found in Europe, South Asia, East Asia, Southeast Asia, and Oceania, where the number of cross-reactive HLA-I SWPs was 1.75–4 times that of WSPs. SWPs with frequencies of greater than 10 are listed in Supplementary Table 10. For HLA-II, CREs could only be detected in North American, Europe, East Asia, and South Asia; 2,206 cross-reactive HLA-II SWPs were detected, which was 4 times the number of WSPs (546). In North America, Europe, and East Asia, the number of cross-reactive HLA-II SWPs was 2.4–19.4 times the number of WSPs. In contrast, we detected 13 cross-reactive HLA-II WSPs in data from South Asia; this was higher than the number of SWPs, possibly because of the lack of sequence data submitted from South Asia. These results indicated that the natural selective pressure caused by pre-existing cross-reactive T-cell immunity may have driven the evolutionary direction of SARS-CoV-2 and allowed the virus to escape immune monitoring. Moreover, to evaluate if this is a random phenomenon, the cross-reactive peptides (CRPs) and all appeared peptides besides CRPs in the whole genome were used for statistical analysis. As been illustrated in Supplementary Tables 5, 6, the WSPs are significantly higher than SWPs in all appeared peptides beside CRPs. For example, 13 times for MHC I, and 4 times for MHC II. On the contrary, the number of SWPs are larger than the number of WSPs, with approximately 14 and 4 times for MHC I and MHC II, respectively. The Pearson’s Chi-squared test showed that the p-values in both tests are less than 2.2e-16, indicating potential natural selection of SARS-CoV-2 by pre-existing cross-reactive T-cell immunity.

## Mutations on S Protein Lead to Increased Binding Capacity to ACE2 and Decreased Affinity to Antibodies

The S protein of SARS-CoV-2 is a major target for humoral immunity, and the receptor-binding domain (RBD) of S protein mediates the attachment of viruses to surface receptors in the host cell (Song et al., 2004; Kirchdoerfer et al., 2016; Schoeman and Fielding, 2019). Also, the mutations on the main antigenic protein of SARS-CoV-2, S protein, were evaluated through binding analysis. Both the ACE2-RBD binding and mAb-RBD binding analysis were provided through protein docking approaches. Results indicated that the mutations on the RBD trend to reduce the binding affinity between mAb and RBD, meanwhile, increase the binding affinity between human ACE2 and RBD. This means the mutations on S protein could not only reduce the antigenicity to the immune system but also increase the infectivity of the virus by increase the binding affinity to human ACE2. Consistently, the latest experiments also indicated that mutations on the RBD may significantly increase the infectivity of SARS-CoV-2, such as the D614G mutations (Li et al., 2020). Here, we evaluated the influence of mutations occurring in the ACE2 binding domain and epitopes regions of S protein. The three-dimensional structures of three mutants with mutations in the ACE2 binding domain, including R408I, N439K, and G476S (Figure 2D), and four mutants with mutations in epitope regions, including K378R, H519P, H519Q, and R408I (Figure 2E), were constructed by homology modeling (Webb and Sali, 2008). Furthermore, the binding affinities between corresponding mutants and ACE2 (Lan et al., 2020) or CR3022 antibody (Yuan et al., 2020) were calculated through molecular docking (Pierce et al., 2014). The RBD region of SARS-CoV-2 isolate (MN908947.3) was selected as a reference for the control.

The mutants R408I, N439K, and G476S promote binding between the RBD region and ACE2 compared with the reference (Figure 2E). Arginine (R) and lysine (K) are alkaline amino acids, and the mutations of R to isoleucine (I) and asparagine (N) to K result in significant changes in the properties of the protein. Further investigation demonstrated that the potential binding site for N439K was the acidic amino acid glutamic acid (329E), which could form an ionic bond to increase the binding capacity between RBD and ACE2 (Figure 2E). Moreover, the potential binding site for R408I was the alkaline amino acid histidine (H), and the mutations R408I may reduce the repulsive force between the two alkaline amino acids H and R (Figure 2E).

The mutations K378R, H519P, H519Q, and R408I can decrease the binding affinity of S protein to the CR3022 antibody (Figure 2E). In addition to the mutation K378R, which is located between alkaline amino acids, the other three mutations were all alkaline amino acids mutated to uncharged residues. Notably, 408R was found to form an ionic bond with the aspartic acid (D) at 54D on the H chain. After R was mutated to I, the ionic bond would be broken, thereby reducing the binding affinity to the antibody. The above results indicated that mutations on the RBD of S protein may enhance the ability of the virus to target the ACE2 receptor and further promote the capacity of the virus to invade host cells. Furthermore, mutations on the RBD could decrease the binding affinity for antibodies and lead to immune escape of the virus.

Moreover, multiple recent studies indicated that the RBD based vaccine could induce T-cell response. For example, in Ugur Sahin’s work (Sahin et al., 2020), the activation of virus-specific CD4+ and CD8+ T-cells were both observed by an nRBD-based COVID-19 vaccine. Also, another study indicated that the magnitude of the T-cell ELISpot response at 6 months against the spike protein was strongly correlated with the magnitude of the peak antibody level against both spike protein and the RBD domain (Zuo et al., 2021). Thus, the linear epitopes in the RBD domain could induce T-cell immunity and the mutations in RBDs could also change the ability to induce T-cell immunity and alter the T-cell responses.

## Discussion

In conclusion, we mapped all observed mutations on the whole genome of each SARS-CoV-2 isolate within the circulating regions to analyze potential natural selection of the virus and related outcomes. The results indicated that long-term and large-scale community transmission in continents such as North America and Europe led to a high mutation frequency in both ORF1ab and S proteins. Thus, circulating SARS-CoV-2 may be under heavy selective pressure of the host immune system, and mutations could cause an increase in the number of WSPs compared with the number of SWPs on the whole genome. However, for CREs between seasonal HCoVs and SARS-CoV-2, the number of SWPs was significantly higher than the number of WSPs on ORF1ab protein, indicating the potential natural selection of SARS-CoV-2 by pre-existing cross-reactive T-cell immunity. Finally, we found that the S protein of SARS-CoV-2 may not only enhance binding affinity with human receptor ACE2 by forming a new ionic bond through mutations, such as N439K, but also reduce the binding affinity of CR3022 antibody by destroying the ionic bond through the mutation R408I. This result suggested that S protein may exhibit an evolutionary trend for increasing infectious ability and escaping immune monitoring under antibody-mediated B-cell immune pressure. According to recent research (Kissler et al., 2020), SARS-CoV-2 is more likely to spread much longer than expected.

Recent studies indicated that mutations may significantly affect the vaccine efficacy against SARS-CoV-2. The viral mutation and recombination events on the S protein could diminish or negate the efficacy of first-generation vaccines (Poland et al., 2020a). Moreover, vaccine development could be obstructed if the mutation on the S protein could evade immunity (Poland et al., 2020b). More evidence such as the mutant South African strains could decrease the protective efficiency in vaccinated humans (Wang et al., 2021). All the above results indicated that the mutations on the SARS-CoV-2, especially in S protein, will hold the potential to decrease the efficiency of currently developed vaccines. Although SARS-CoV-2 and influenza virus are both RNA viruses and depend on the viral RNA polymerase to express their proteins, SARS-CoV-2 has a proofreading mechanism that makes the mutation rate slower than the influenza virus. Thus, the vaccine of SARS-CoV2, as well as the immunity developed in recovered patients, could provide longer lasting protection than influenza virus (Manzanares-Meza and Medina-Contreras, 2020). However, according to the current best knowledge, it is virtually certain that further mutations and even recombination events will be identified (Poland et al., 2020a), it is still a great challenge to develop protective SARS-CoV-2 vaccines or we may need to renew the SARS-CoV-2 vaccines periodically like the influenza virus.

This research suggests that if immune memory is only effective in the short-term, there could be risk of annual or periodic outbreaks every 2–3 years, similar to seasonal HCoVs (Kissler et al., 2020). Consistent with this, our research indicated that rapid mutations in SARS-CoV-2 may tend to escape from the monitoring and recognition of the immune system. This suggests that even if an effective vaccine can be developed for the current circulating SARS-CoV-2, mutations promoting rapid immune escape may make any vaccine ineffective within a short time. Thus, we suggest that vaccine development for SARS-CoV-2 may be cyclical, similar to influenza virus. Under such circumstances, monitoring of mutations and the antigenic evolution of SARS-CoV-2 will be necessary.

## Data Availability Statement

The original contributions presented in the study are included in the article/Supplementary Material, further inquiries can be directed to the corresponding author.

## Author Contributions

TQ, QL, and CZ designed the experiments. CZ and XJ collected the data and performed the analysis. XJ, XC, and LQ organized the results and contributed to data interpretation. TQ and QL co-supervised the whole project. All authors contributed to the wrote of the manuscript.

## Conflict of Interest

The authors declare that the research was conducted in the absence of any commercial or financial relationships that could be construed as a potential conflict of interest.
