# Supplementary material for: Antigenic Evolution on a Global Scale Reveals the Potential Natural Selection of Severe Acute Respiratory Syndrome-Coronavirus 2 by Pre-existing Cross-Reactive T-Cell Immunity
Source: Front Microbiol. 2021 May 18;12:599562. doi: 10.3389/fmicb.2021.599562 (PMC8169977; doi:10.3389/fmicb.2021.599562)
Supplement: Supplementary file 1 [file Data_Sheet_1.DOCX]

**Supplementary Methods**

**Data source**

The reference amino acid sequences of SARS-CoV-2 (MN908947.3) and four human susceptible seasonal CoVs including HCoV-229E (NC_002645.1), HCoV-HKU1 (NC_006577.2), HCoV-NL63 (NC_005831.2), and HCoV-OC43 (NC_006213.1) were derived from the National Center for Biotechnology Information (NCBI) [^1^](#_ENREF_1).

The protein sequences of SARS-CoV-2 strains were retrieved from the National Genomics Data Center (NGDC) [^2^](#_ENREF_2) till May 20^th^, 2020. Only those sequences that have passed the high-quality assessment (reference the NGDC website) were included in this study, which left 15,183 distinct strains. The available mutation annotation information (GFF3 files) for all the strains were downloaded from NGDC. Then, 10 structure and non-structure proteins including spike protein, envelope protein, nucleocapsid protein, membrane protein, orf1ab protein, ORF3a, OFR6, ORF7a, ORF8, and ORF10 were derived from the GFF3 annotation files. For mutation analysis of the whole protein sequence, we extended the indel positions to the reference sequence to obtain a more precise mutation frequency for each amino acid point. For ease of k-mer analysis, all non-mismatch mutations are excluded, which left 13,432 strains and 4,420 mutation sites for downstream analysis.

Frequencies of alleles in different countries and regions were obtained from the Allele Frequency Net Database (AFND) [^3^](#_ENREF_3). For each country and region, only those alleles with frequency over 0.05 and population over 25 were retained.

**Peptide for epitope prediction**

***Mutant peptide.*** For each mutation site $i$ compared with reference sequence Wuhan-Hu-1(MN908947.3), the sequence from site $i-15$ to $i+15$ was derived from the corresponding protein sequence of the mutants. Further, it will be divided into peptides with the sliding windows of 8-mer to 15-mer and searching step of 1-mer. Among all peptides, those involving the mutation site $i$ was retained as the mutant epitopes.

***Cross-reactive peptide.*** Each of the mutant peptide of SARS-CoV-2 was compared against all four seasonal CoVs using in-house python script. The cross-reactive epitopes were defined as peptides that are identical to the k-mer sequence in at least one of the seasonal CoVs.

**Prediction of HLA binding affinity**

Both the HLA-I and HLA-II binding affinities between HLA alleles and k-mer peptides were predicted by the T Cell Epitope Prediction Tools (standalone version 2.22.3) with IEDB (Immune Epitope Database) recommended methods[^4^](#_ENREF_4)^,^ [^5^](#_ENREF_5). For HLA I alleles, 8-14 mer peptides were analyzed and considered strong binding peptides with a cutoff ≤ 0.01[^6^](#_ENREF_6). For HLA II alleles, only 15 mer peptides were analyzed and a consensus percentile rank of the top 10% was considered strong binding peptide[^7^](#_ENREF_7). The allele-peptide pairs with prediction score lager than the cutoff was considered weak binding peptides. The total number of strong or weak binders were determined by the combination of strains and alleles.

**Analyze the amino acid mutations on S protein**

***Mapping the mutation sites on ACE2 binding sites and epitope residues.*** The ACE2 binding sites on the receptor binding domain (RBD) of S protein were derived from crystalized structure (PDB id: 6vw1). The epitope residues on S protein RBD were derived from the RBD-antibody complex (PDB id: 6w41). The threshold of interaction-interface residues from each complex were defined as the residues with nearest atom distance less than 6Å. Then, the amino acid mutations can be mapped on ACE2 binding sites and epitope residues.

***Binding affinity prediction.*** For each mutant, the binding affinity towards the ACE2 or CR3022 was predicted through ZDOCK[^8^](#_ENREF_8). The original complex was also divided for molecular docking as reference. The binding affinity was defined as the output ZDOCK score for top 1 predicted complex structure.

**Supplementary Table**

**Supplementary Table 1. Mutation Frequency on the whole genome of SARS-CoV-2^*^.**

^*^Each line illustrated the protein name, mutation positions, mutation counts, mutation frequency and whether is it in the conserved regions.

**Supplementary Table 2. Top ranking mismatch mutations on the whole genome of SARS-CoV-2^*^.**

^*^Each line illustrated the protein name, mutation positions, number of mutation types, mutation counts and counts of different mutation types.

**Supplementary Table 3. Top ranking mutations on the circulating strains in different regions^*^.**

^*^Each lines illustrated the protein name, mutation positions, mutation types, circulating countries, mutation counts and circulating continent.

**Supplementary Table 4. The immunogenicity of epitopes in ORF1ab protein*.**

^*Here, we adopt analytical method provided by IEDB Class I Immunogenicity to predicted the epitopes in ORF1ab*^

**Supplementary Table 5. Numbers of four types of HLA-I PTEs before and after the mutations**^*^**.**

*Here, we defined four types of predicted HLA-I PTEs. The predicted binding affinity was defined as strong or weak according to defaulted threshold before or after the mutations. For example, strong🡪weak refers to the peptide predicted as strong binding before the mutation and alter to weak binding after the mutation.

**Supplementary Table 6. Numbers of four types of HLA-II PTEs before and after the mutations**^*^**.**

*Here, we defined four types of predicted HLA-II PTEs. The predicted binding affinity was defined as strong or weak according to defaulted threshold before or after the mutations. For example, strong🡪weak refers to the peptide predicted as strong binding before the mutation and alter to weak binding after the mutation.

**Supplementary Table 7. Number of all epitopes identified in Matenus’s work*.**

^*Here, we calculated the number of all epitopes identified in Matenus’s work*.^

**Supplementary Table 8. The major HLA types and top mutations in global regions analyzed in our study*.**

^*Here, we provide the major HLA types and top mutations in global regions analyzed in our study*.^

**Supplementary Table 9. Numbers of SWPs and WSPs cross-reactive peptide between seasonal HCoVs and SARS-CoV-2**^*^**.**

*Here, we provide the available number of weak to strong peptides (WSPs) and strong to weak peptide (SWPs).

**Supplementary Table 10. SWPs of CRPs between seasonal HCoVs and SARS-CoV-2**^*^**.**

*Here, we presented all SWPs with counting number of 10 in our dataset. Each line represents the protein name, peptides on the reference protein, peptides on the mutants, mutations, start and end positions on the whole genome.

**Reference**

1 Dooley, E. E. National Center for Biotechnology Information. *Environmental health perspectives* **112**, A674, doi:10.1289/ehp.112-1277128 (2004).

2 National Genomics Data Center, M. & Partners. Database Resources of the National Genomics Data Center in 2020. *Nucleic acids research* **48**, D24-D33, doi:10.1093/nar/gkz913 (2020).

3 Gonzalez-Galarza, F. F. *et al.* Allele frequency net database (AFND) 2020 update: gold-standard data classification, open access genotype data and new query tools. *Nucleic acids research* **48**, D783-D788, doi:10.1093/nar/gkz1029 (2020).

4 Wang, P. *et al.* A systematic assessment of MHC class II peptide binding predictions and evaluation of a consensus approach. *Plos Comput Biol* **4**, e1000048, doi:10.1371/journal.pcbi.1000048 (2008).

5 Moutaftsi, M. *et al.* A consensus epitope prediction approach identifies the breadth of murine T(CD8+)-cell responses to vaccinia virus. *Nature biotechnology* **24**, 817-819, doi:10.1038/nbt1215 (2006).

6 Jurtz, V. *et al.* NetMHCpan-4.0: Improved Peptide-MHC Class I Interaction Predictions Integrating Eluted Ligand and Peptide Binding Affinity Data. *Journal of immunology* **199**, 3360-3368, doi:10.4049/jimmunol.1700893 (2017).

7 Paul, S. *et al.* HLA class I alleles are associated with peptide-binding repertoires of different size, affinity, and immunogenicity. *Journal of immunology* **191**, 5831-5839, doi:10.4049/jimmunol.1302101 (2013).

8 Pierce, B. G. *et al.* ZDOCK server: interactive docking prediction of protein-protein complexes and symmetric multimers. *Bioinformatics* **30**, 1771-1773, doi:10.1093/bioinformatics/btu097 (2014).
